# Up-regulation of microRNA-200c-3p inhibits invasion and migration of renal cell carcinoma cells via the SOX2-dependent Wnt/β-catenin signaling pathway

**DOI:** 10.1186/s12935-019-0944-5

**Published:** 2019-09-07

**Authors:** Shuai Li, Ziyu Feng, Xuechong Zhang, Dongyang Lan, Yudong Wu

**Affiliations:** Department of Urology, The First Affiliated Hospital of Zhengzhou University, No. 1 Longhu Central Ring Road, Zhengzhou, 450052 People’s Republic of China

**Keywords:** microRNA-200c-3p, Renal cell carcinoma, SOX2, Wnt/β-catenin signaling pathway, Proliferation, Invasion, Migration, Apoptosis

## Abstract

**Background:**

MicroRNA-200c-3p (miR-200c-3p) has been revealed to be related to renal cell carcinoma (RCC) progression, while the inner mechanisms remain unknown. In our study, we intend to unearth the capability of miR-200c-3p in RCC development via the Wnt/β-catenin signaling pathway through binding to SOX2.

**Methods:**

miR-200c-3p, SOX2, β-catenin and GSK3β expression in both tissues and cells of RCC were detected by RT-qPCR or western blot analysis. miR-200c-3p was restored or silenced to determine their biological functions of RCC cells. Expression of SOX2 and related proteins in the Wnt/β-catenin signaling pathway were evaluated by RT-qPCR and western blot analysis. The effect of the combination of downregulated miR-200c-3p and downregulated SOX2 on cell biological behavior change was also determined.

**Results:**

Initially, we found that miR-200c-3p was declined while SOX2, β-catenin and GSK3β was elevated in RCC tissues and cells. A498 cells with the largest difference in miR-200c-3p expression and OS-RC-2 cells with the smallest difference were selected for subsequent experiments. Additionally, upregulated miR-200c-3p and downregulated SOX2 was determined to suppress proliferation, migration, invasion and induce apoptosis of RCC cells. Furthermore, miR-200c-3p inhibited SOX2 to inactivate the Wnt/β-catenin signaling pathway.

**Conclusion:**

Collectively, this study highlights that upregulated miR-200c-3p inhibits expression of SOX2, thereby inhibiting development of RCC cells via modulating the Wnt/β-catenin signaling pathway activation.

## Background

Renal cell carcinoma (RCC) is the 3th most frequent urological cancer be next only to prostate cancer and bladder cancer, and RCC accounts for around 3% adult malignancies and exceeds 90% of neoplasms resulting from the kidney [1]. The recognized risk factors of RCC include male sex, increasing age, smoking, as well as genetic predisposition, while little consensus reaches regarding other RCC risk factors [2]. Due to the short of diagnosis biomarkers and particular symptoms at an early stage, approximately 20–30% of RCC patients have had metastasis at the time of initial diagnosis [3]. Despite surgery is considered to be curative for localized disease, a larger number of RCC patients develop metastases or relapses, which are related to poor prognosis [4]. Therefore, it is essential to realize the molecular mechanisms in RCC recurrence and metastasis, for the reason that this knowledge may offer help to improve RCC treatment.

The microRNA-200 (miR-200) family containing miR-200a/b/c, − 141 as well as − 429 has been demonstrated to exert functions in epithelial mesenchymal transition (EMT) [5]. Increasing researches have indicated that the decreased miR-200 family members is found in human cancers and among which, miR-200c is detected to be down-regulated in RCC [6–8]. Chang et al. have proposed that miR-200c may be considered as potential biomarkers and also, help to provide alternative options for RCC treatment [9]. MiRNAs are able to modulate mRNA expression in a tissue specific approach, either by suppressing translation or transcription or stimulating degradation of the transcript [8]. The family of SRY (sex determining region Y)-box (SOX) is a group of transcription factors with great importance in cancer development and stem cell biology [10]. SOX2, one of the SOX family members, is a primary regulator of neural competence in both human and vertebrates [11]. Evidence has shown that SOX2 has emphasized its vital role in stem cell maintenance, which is a lineage fate determinant together with a necessary part to reprogram the pluripotency of somatic cells [12, 13]. SOX2 is reported as either oncogene or tumor suppressor gene in various cancer types. SOX2 is found to be upregulated in colon cancer, small cell lung cancer and esophageal squamous cell carcinoma [14–16]. However, SOX2 could also acts as tumor suppressor gene in several caners, such as gastric cancer [17], which further highlights the context-specific characteristics of SOX involvement in carcinogenesis. It has been reported that high SOX2 expression is related to poor prognosis for RCC, implying its role of oncogene in RCC [18]. On the contrary, Liu et al. have found that SOX2 expression level was significantly declined in RCC, suggesting its inhibitory role in RCC [19]. No consensus has been reached upon this issue. As previously described, SOX proteins physically bind with β-catenin to control the expression of target genes of Wnt [13]. A study has suggested that the Wnt/β-catenin signaling pathway has a close association with many types of human carcinomas including RCC [20, 21]. Based on these evidence, we speculate that miR-200c-3p inhibits development of RCC cells via the SOX2/Wnt/β-catenin axis.

## Materials and methods

### Ethics statement

All the specimens in this study were collected with the informed consent of the patients, and this study was approved by the ethics committee of the First Affiliated Hospital of Zhengzhou University.

### Clinical sample collection

Human RCC tissues and adjacent normal tissues were acquired from 56 patients with RCC that were enrolled into the First Affiliated Hospital of Zhengzhou University from September 2014 to October 2017. The pathological data of all clinical cases were complete and confirmed by clinical, imaging and pathology. Among these 56 patients, there were 47 males and 9 females, with the mean age of 56 years old. The patients excluded major basic diseases such as heart, liver and lung diseases, and they didn’t receive chemotherapy or targeted drugs and other treatment measures before operation. Each tissue specimen was frozen in liquid nitrogen.

### Cell culture

Human RCC cell lines OS-RC-2, G401, A498, Caki-1 and ACHN cells and human embryonic kidney cells HEK-293 were purchased from American Type Culture Collection (ATCC, USA). The cells, together with the cryopreserved tube, were immediately placed at 37 °C so that the cells could be resuscitated and melted as soon as possible. The melted cells were absorbed from the ultra-clean workbench and added to the centrifuge tube, and the centrifuge tube was added with calf serum-free RPMI 1640 medium (PM150110, Procell LifeScience & Technology Co. Ltd., Wuhan, China). The cells were suspended and then centrifuged for 1500 rpm for 5 min. After the supernatant was removed, the cells were added with cell culture medium containing 15% fetal bovine serum (FBS). The cells were inoculated in a culture bottle and placed in a 37 °C incubator containing 5% CO_2_. The cells were subcultured routinely and the growth logarithmic cells were used in the experiment.

### Cell transfection and grouping

OS-RC-2 and A498 (2 × 10^5^ cells/well) during logarithmic growth period were inoculated into a 6-well cell culture plate. The cells were transfected when the cell adhered to the wall and the cell confluence reached 30–50%. The cell transfection was conduced based on the instructions of the lipofectamine 2000 kit (11668-027, Invitrogen, Carlsbad, California, USA). After transfection, the cells were cultured at 37 °C with 5% CO_2_ and saturated humidity. After 4–6 h, the medium containing the transfection solution in the well was discarded and replaced with RPMI 1640 medium containing 10% FBS (PM150110, Wuhan Punosei Life Technology Co., Ltd., Wuhan, China). After 24 to 48 h, it was used for subsequent experiments.

OS-RC-2 cells were transfected with miR-200c-3p-mimics negative control (NC) or miR-200c-3p-mimics (miRNA mimic is a mimic synthesized by chemical methods, which can mimic the high level expression of mature miRNAs in cells, so as to enhance the regulation of endogenous miRNAs). A498 cells were introduced with miR-200c-3p-inhibitors NC, miR-200c-3p-inhibitors, si-SOX2 or miR-200c-3p-inhibitors + si-SOX2 sequence. All these sequences were purchased from Shanghai GenePharma Co., Ltd (Shanghai, China).

### Reverse transcription quantitative polymerase chain reaction (RT-PCR)

Trizol (15596-018, Invitrogen, Carlsbad, California, USA) was employed for extracting the total RNA of tissues and cells. Diethylpyrocarbonate (DEPC, A100174-0005, Shanghai Sangon Biotechnology Co., Ltd., Shanghai, China) treated with ultrapure water was utilized to dissolve RNA. The absorbance at 260 nm and 280 nm was measured using an ND-1000 UV/Vis spectrophotometer (Thermo Scientific, MA, USA) to identify the mass of total RNA and adjust the RNA concentration. The extracted RNA was subjected to reverse transcription in a two-step method according to the kit (Thermo Scientific, MA, USA). RT-qPCR was performed by TaqMan probe method, and the reaction system was operated on the basis of the instructions of the kit (KR011A1, Beijing Puyihua Technology Co., Ltd., Beijing, China). The primer sequences are shown in Table 1. PCR was performed via ABI 7900 Real time-PCR instrument (Bio-Rad iQ5, San Francisco, USA). U6 was used as the internal control of miR-200-3c, while glyceraldehyde phosphate dehydrogenase (GAPDH), internal control of other factors. The target gene expression was calculated by 2^−∆∆Ct^ method [13].

**Table 1:** Primer sequence

Gene	Forward	Reverse
miR-200c-3p	5′-GGGAACACACCTGGTTAAC-3′	5′-CAGTGCGTGTCGTGGAGT-3′
U6	5′-TGCGGGTGCTCGCTTCGGCAGCA-3′	5′-CCACTGCAGGGTCCGAGGT-3′
SOX2	5′-CGCCCCCAGCAGACTTCACA-3′	5′-CTCCTCTTTTGCACCCCTC-3′
β-catenin	5′-ATGGCTTGGAATGAGAC-3′	5′-AACTGGATAGTCAGCACC-3′
GSK3β	5′-CCTTAACCTGGTGCTGGACT-3′	5′-AGCTCTGGTGCCCTGTAGTA-3′
GAPDH	5′-ACCACAGTCCATGCCATCAC-3′	5′-TCCACCACCCTGTTGCTGTA-3′

*miR-200c-3p* microRNA-200c-3p, *GAPDH* glyceraldehyde phosphate dehydrogenase

### Western blot analysis

Cells in each group were collected in a centrifuge tube and added with 100 μL of radioimmunoprecipitation assay lysate (R0020, Beijing Solarbio Technology Co., Ltd., Beijing, China) (containing 1 mmol/L phenylmethyl sulfonylfluoride, currently used), and homogenize at 3000 r/min. The proteins were extracted and the protein concentration was evaluated in view of the protocols of the bicinchoninic acid assay (AR0146, Boster, Wuhan, China). Following 10% sodium dodecyl sulfate polyacrylamide gel electrophoresis separation, protein samples were next transferred onto a polyvinylidene fluoride membrane (P2438, Sigma-Aldrich, St. Louis, Missouri, USA). Afterwards, the membrane was sealed with 5% bovine serum albumin and appended with the primary antibodies against β-catenin (ab3927, 1:1000), GSK3β (ab86714, 1:1000) and GADPH (ab181602, 1:10,000 (Abcam, Cambridge, MA, USA), followed by the anti-rat secondary antibody (ab6789, 1:2000, Abcam, Cambridge, MA, USA), and an enhanced chemiluminescence solution together with Bio-rad Gel Doc EZ imager (Bio-rad, California, USA) were utilized for developing. The gray value analysis of target band was analyzed by Image J software.

### Bioinformatics analysis and dual luciferase reporter gene assay

Online website (http://www.targetscan.org) was employed to predict the binding between miR-200c-3p and SOX2. The human target gene sequence was queried in GenBank (National Center for Biotechnology Information, Bethesda, Maryland, USA) and a 3′-untranslated region (UTR) sequence containing the miR-200c-3p potential target gene SOX2 was design based on the predicted results of the software. A plasmid vector containing the SOX2-3′UTR wild-type (WT) and SOX2-3′UTR mutant type (MUT) reporter gene was constructed using the site-directed mutation technique. The cells were co-transfected with SRX2-WT and SOX2-MUT plasmids for 24 h with miR-200c-3p mimics NC and miR-200c-3p mimics, respectively. The medium was renewed and continued to culture for 48 h to lyse the cells. The luciferase activity was detected by a luminometer (TD20/20, Turner Designs, Sunnyvale, CA, USA) among with a luciferase detection kit (E1910, Inner Mongolia HengSeng Biotechnology Co., Ltd., Inner Mongolia, China).

### Cell counting kit-8 (CCK-8) assay

At 48 h post transfection, the cells were collected and detached with 0.25% trypsin. The cell suspensions of each group were diluted with a certain concentration and then inoculated into 96-well plates at the density of 5 × 10^4^ cells/mL. Each well was added with 10 μL cell culture medium. The optical density (OD) value at zero time point was measured at first, and then measured every 24 h, namely 24 h, 48 h, 72 h. Subsequently, each well was appended with 10 μL CCK-8 solution (Beyotime Biotechnology, Shanghai, China) and incubated at 37 °C for 2 h. The OD value of each well was measured at the wavelength of 430 nm by a microplate reader (Beijing Jingke Ruida Technology Co., Ltd., Beijing, China). Each reaction was run in triplicate.

### Flow cytometry

At 48 h post transfection, the trypsin-detached cells in each group were harvested and centrifuged, and then the supernatant was discarded. Subsequently, the cells were suspended and washed with phosphate buffer saline (PBS), thus the single cell suspension was prepared. The single cell suspension was centrifuged for 5 min at 1000 rpm, and the supernatant was removed. The cells were washed with PBS two times and fixed with 70% ethanol for 30 min. After that, the centrifuged cells were washed with PBS two times and appended with 1% propidium iodide (PI) containing RNA enzyme. After being stained for 30 min, the cells were washed with PBS two times to remove PI. Finally, the cell cycle distribution was determined by a BD-Aria Flow Cytometer (FACSCalibur, Beckman Coulter, USA).

After the cells were detached with trypsin without ethylene diamine tetraacetic acid, the suspension cells were centrifuged to collect the cells, with the supernatant discarded. According to the Annexin-V-fluorescein isothiocyanate (FITC) Apoptosis Detection Kit (C1065, Beyotime Biotechnology, Shanghai, China), the Annexin-V-FITC/PI dye solution was formulated with Annexin-V-FITC, PI, hydroxyethyl piperazine ethanesulfonic acid (HEPES) buffer solution in a ratio of 1:2:50. About 1 × 10^6^ cells were suspended with 100 μL dye solution and appended with 1 mL HEPES buffer solution after 15-min incubation. A flow cytometer at 488 nm excitation wavelength, with a 525 nm or 620 band-pass filter, was utilized for FITC or PI fluorescein detection.

### Transwell assay

Matrigel (40111ES08, Yeasen, Shanghai, China) was dissolved at 4 °C overnight. Matrigel was diluted 1:3 in serum-free DMEM medium for three times (15 μL, 7.5 μL, 7.5 μL), and 30 μL of diluted Matrigel was added to the apical chamber of each Transwell chamber. At 10 min intervals, Matrigel was evenly spread and covered with all the microwells on the underside of the apical chamber. At 48 h after transfection, cells were collected to prepare cell suspension. The cells were seeded in Transwell apical chamber, and then supplemented with 0.5 mL DMEM medium containing 10% FBS. Next, the cells were added to basolateral chamber of the 24-well plate and placed in an incubator at 37 °C with 5% CO_2_. After 48 h of incubation, the unpenetrated cells in the apical chamber were gently wiped off with cotton swabs. The membrane was fixed in 95% ethanol for 15–20 min, washed with water and then stained with methyl violet for 10 min, washed again with water, and observed under a high-inverted microscope. The average of 5 high-field cell counts was taken for each sample. The number of cells passing through Matrigel was an indicator of their invasive ability.

### Scratch test

After 48 h of different treatment, the cells were collected and inoculated into a 6-well plate at a cell density of 1 × 10^5^ cells/well. When the cell confluence reached 90%, 4 traces were drawn with 200 μL tip heads. Image J software was used to analyze the migration ability of cells in each group. The experiment was repeated three times.

### Statistical analysis

All the data were analyzed with statistical SPSS 21.0 (IBM Corp. Armonk, NY, USA) software. The measurement data in normal distribution (tested by the Kolmogorov–Smirnov test) were depicted as mean ± standard deviation. Comparison between two groups was analyzed by the t test, and among multiple groups, by one-way analysis of variance (ANOVA). The Fisher’s least significant difference t test (LSD-t) was applied for pairwise comparison. *P* value ≤ 0.05 indicative of statistically significant.

## Results

### Downregulated miR-200c-3p and upregulated SOX2, β-catenin and GSK3β are found in RCC tissues

**Fig. 1:**
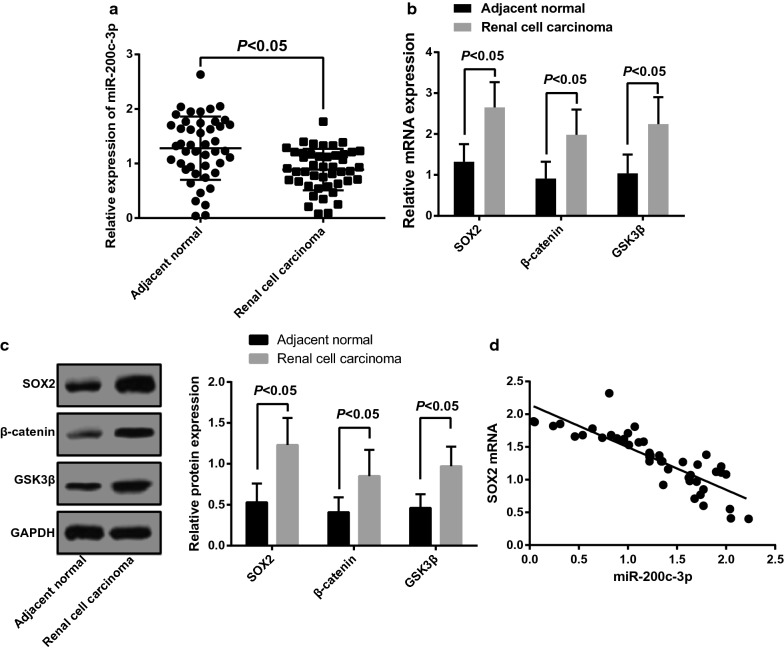
Expression of miR-200c-3p, SOX2, β-catenin and GSK3β in RCC tissues and adjacent normal tissues. **a** Expression of miR-200c-3p in RCC tissues and adjacent normal tissues. **b** Expression of SOX2, β-catenin and GSK3β mRNA in RCC tissues and adjacent normal tissues. **c** Expression of SOX2, β-catenin and GSK3β protein in RCC tissues and adjacent normal tissues. **d** Correlation analysis of miR-200c-3p expression and SOX2 mRNA expression. N = 56

RT-qPCR was used to detect miR-200c-3p expression in RCC tissues and corresponding adjacent normal tissues. The obtained results highlighted that the poor expression of miR-200c-3p was found in RCC tissues relative to that in adjacent normal tissues, as shown in Fig. 1a. Meanwhile, the expression of SOX2, β-catenin and GSK3β in RCC tissues and corresponding adjacent normal tissues was also determined by RT-qPCR and western blot analysis. The findings suggested the in contrast to adjacent normal tissues, the expression of SOX2, β-catenin and GSK3β was increased in RCC tissues (all *P* < 0.05; Fig. 1b, c). Besides, the relationship between miR-200c-3p expression and SOX2 mRNA expression was analyzed by Pearson correlation analysis. The results showed that (Fig. 1d) the miR-200c-3p expression was negatively correlated with SOX2 mRNA expression (r = − 0.850, *P* < 0.001).

### Downregulated miR-200c-3p and upregulated SOX2 are found in RCC cells

**Fig. 2:**
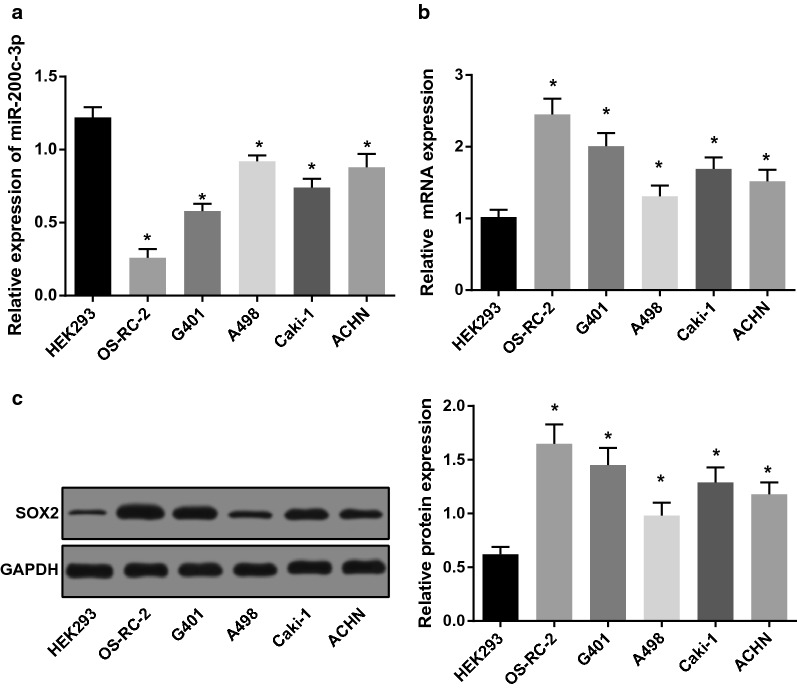
Expression of miR-200c-3p and SOX2 in RCC cells and human embryonic kidney cells. **a** Expression of miR-200c-3p in RCC cells and human embryonic kidney cells. **b**, **c** Expression of SOX2 mRNA and protein in RCC cells and human embryonic kidney cells. N = 5. *P < 0.05 vs normal embryonic kidney cells (HEK-293)

miR-200c-3p expression in RCC cells (OS-RC-2, G401, A498, Caki-1 and ACHN cells) and human embryonic kidney cells HEK-293 was detected by RT-qPCR. As presented in Fig. 2a, the results suggested that compared with normal embryonic kidney cells (HEK-293), miR-200c-3p expression in OS-RC-2, G401, A498, Caki-1 and ACHN cell lines were significantly down-regulated. Among them, the down-regulation of miR-200c-3p in A498 cells was the smallest, while that in OS-RC-2 cells was the largest. Meanwhile, the expression of SOX2, β-catenin and GSK3β in RCC cells and HEK-293 cells was also determined by RT-qPCR and western blot analysis. The results suggested that relative to normal embryonic kidney cells (HEK-293), the SOX2 mRNA and protein expression levels elevated in OS-RC-2, G401, A498, Caki-1 and ACHN cells (*P* < 0.05; Fig. 2b, c).

### Expression level of miR-200c-3p and SOX2 in each group

**Fig. 3:**
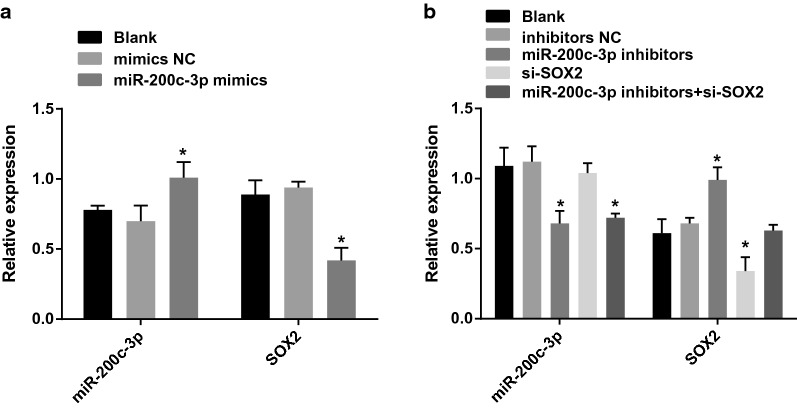
Expression of miR-200c-3p and SOX2 in each group. **a** Expression of miR-200c-3p and SOX2 after miR-200c-3p-mimics transfection into OS-RC-2 cells. **b** Expression of miR-200c-3p and SOX2 after miR-200c-3p-inhibitors and si-SOX2 transfection into A498 cells; N = 5; *P < 0.05 vs the blank group

According to the results of RT-qPCR, we found that in OS-RC-2 cells, the expression of miR-200c-3p elevated and expression of SOX2 mRNA declined in cells upon miR-200c-3p-mimics treatment (both *P* < 0.05). No obvious difference was witnessed in miR-200c-3p expression and SOX2 mRNA expression between the blank group and the miR-200c-3p-mimics NC group (*P* > 0.05) (Fig. 3a).

In 498 cells, no significance was found in miR-200c-3p expression in cells without treatment, cells transfected with miR-200c-3p-inhibitors NC sequence and si-SOX2 sequence (*P* > 0.05). In cells introduced with miR-200c-3p-inhibitors and miR-200c-3p-inhibitors + si-SOX2, there showed decreased miR-200c-3p expression (*P* < 0.05). In 498 cells, no significance was found in SOX2 mRNA expression in cells without treatment, and cells transfected with miR-200c-3p-inhibitors NC sequence and miR-200c-3p-inhibitors + si-SOX2 sequence (*P* > 0.05). In cells introduced with miR-200c-3p-inhibitors, there showed increased SOX2 mRNA expression, while presented decreased SOX2 mRNA expression upon si-SOX2 treatment (both *P* < 0.05) (Fig. 3b).

### miR-200c-3p inhibits expression of SOX2 and inactivates the Wnt/β-catenin pathway

**Fig. 4:**
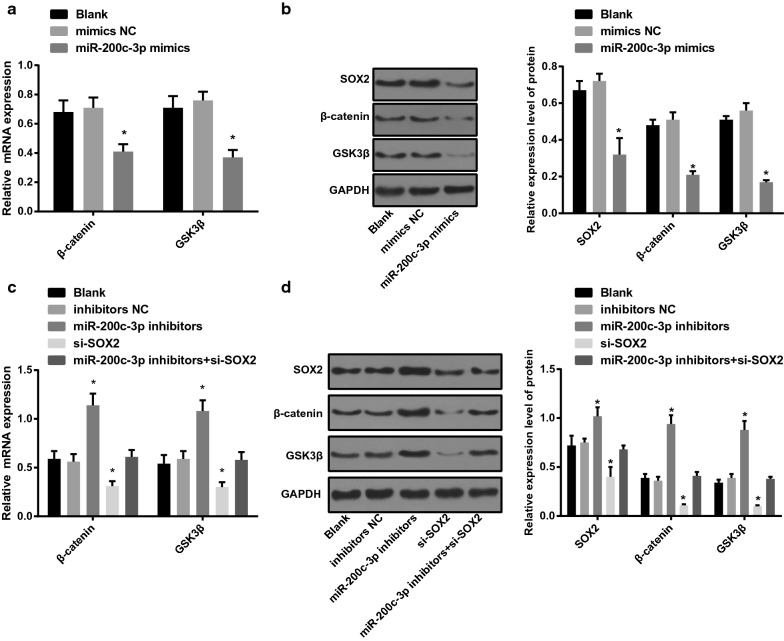
miR-200c-3p inhibits expression of SOX2 and inactivates the Wnt/β-catenin pathway. **a** Expression of β-catenin and GSK3β mRNA after miR-200c-3p-mimics transfection into OS-RC-2 cells. **b** Expression of SOX2, β-catenin and GSK3β protein after miR-200c-3p-mimics transfection into OS-RC-2 cells. **c** Expression of β-catenin and GSK3β mRNA after miR-200c-3p-mimics transfection into A498 cells. **d** Expression of SOX2, β-catenin and GSK3β protein after miR-200c-3p-inhibitors and si-SOX2 transfection into A498 cells; N = 5; *P < 0.05 vs the blank group

As shown in Fig. 4a, b, the findings of RT-qPCR and western blot analysis suggested that expression of SOX2, β-catenin and GSK3β declined in OS-RC-2 cells transfected with miR-200c-3p-mimics sequence (*P* < 0.05). No obvious difference was witnessed in expression of SOX2, β-catenin and GSK3β between the blank group and the miR-200c-3p-mimics NC group (all *P* > 0.05).

Meanwhile, as shown in Fig. 4c, d, there was no difference in expression of SOX2, β-catenin and GSK3β in A498 cells without treatment, miR-200c-3p-inhibitors NC sequence and miR-200c-3p-inhibitors + si-SOX2 sequence (*P* > 0.05). In cells introduced with miR-200c-3p-inhibitors, there showed increased expression of SOX2, β-catenin and GSK3β, while presented decreased expression upon si-SOX2 treatment (all *P* < 0.05).

### miR-200c-3p binds to SOX2 3′-UTR

**Fig. 5:**
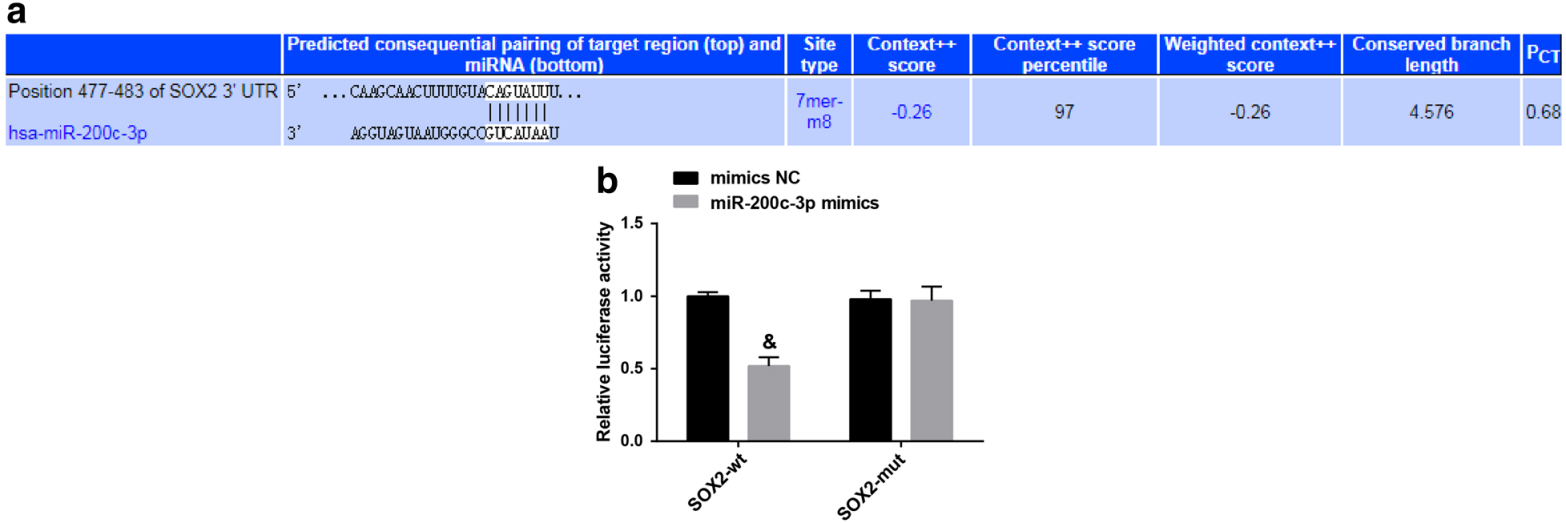
miR-200c-3p binds to SOX2. **a** miR-200c-3p binds to the sequence of the SOX2 3′-UTR region. **b** Luciferase activity assay for verify the relationship between miR-200c-3p and SOX2. *P < 0.05 vs the NC group

Using the biological prediction website http://www.targetscan.org analysis, there was a specific binding region between the 3′UTR of the SOX2 gene and the miR-200c-3p sequence, and SOX2 was determined as the target gene of miR-200c-3p (Fig. 5a). Besides, luciferase activity assay implied that the luciferase activity of the SOX2-WT 3′UTR was suppressed by miR-200c-3p (*P* < 0.05), while the luciferase activity of the SOX2-MUT 3′UTR was not restricted (*P* > 0.05; Fig. 5b).

### Upregulation of miR-200c-3p and downregulation of SOX2 suppress proliferation of RCC cells

**Fig. 6:**
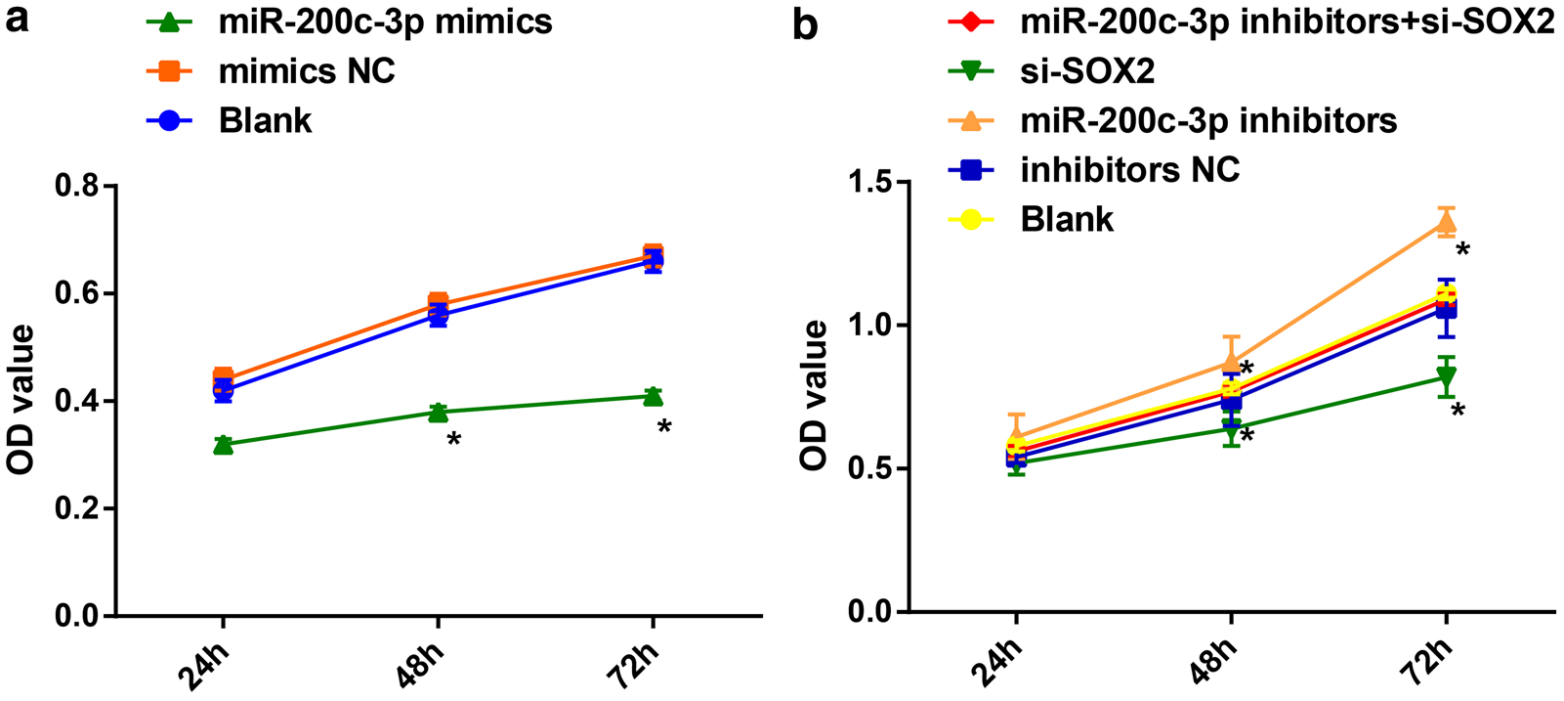
Upregulation of miR-200c-3p suppresses proliferation of RCC cells. **a** Effect of overexpressed miR-200c-3p on OS-RC-2 cell proliferation in each group; **b** Effect of downregulated miR-200c-3p and downregulated SOX2 on A498 cell proliferation in each group. N = 5; *P < 0.05 vs the blank group

The proliferation of OS-RC-2 cells and A498 cells were determined by CCK-8 assay (Fig. 6a, b). The cell growth rate was reduced in OS-RC-2 cells introduced with miR-200c-3p-mimics sequence at 48 h and 72 h, and the OD value was also declined (*P* < 0.05). Meanwhile, there was no difference in cell growth rate in A498 cells without treatment, miR-200c-3p-inhibitors NC sequence and miR-200c-3p-inhibitors + si-SOX2 sequence (*P* > 0.05). In cells in response to miR-200c-3p-inhibitors, there showed increased cell growth rate, while presented decreased cell growth rate upon si-SOX2 treatment (all *P* < 0.05).

### Upregulation of miR-200c-3p and downregulation of SOX2 restrict cell cycle progression and stimulate apoptosis of RCC cells

**Fig. 7:**
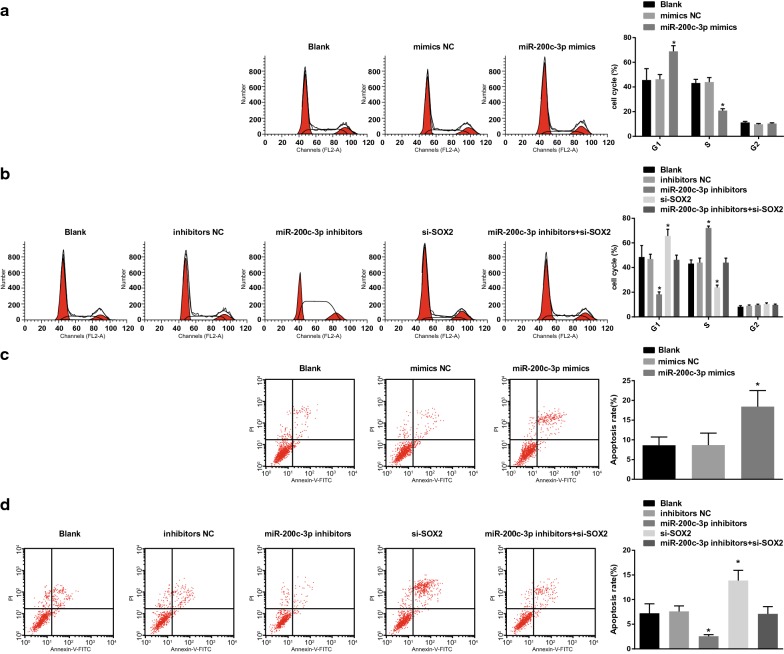
Upregulation of miR-200c-3p restricts cell cycle progression and induces apoptosis of RCC cells. **a** Cell cycle distribution after miR-200c-3p-mimics transfection into OS-RC-2 cells. **b** Cell cycle distribution after miR-200c-3p-inhibitors and si-SOX2 transfection into A498 cells. **c** Cell apoptosis after miR-200c-3p-mimics transfection into OS-RC-2 cells. **d** Cell apoptosis after miR-200c-3p-inhibitors and si-SOX2 transfection into A498 cells. N = 5; * *P* < 0.05 vs the blank group

Based on the results of PI single staining and Annexin V/PI double staining (Fig. 7a–d), we found that in OS-RC-2 cells, no difference was found in proportion of cells in G1, S, and G2 phases together with cell apoptosis in cells without treatment and in cells treated with miR-200c-3p-mimics NC sequence (*P* > 0.05). More cells arrested at G1 phrase and fewer arrested in S phrase as well as increased apoptosis rate exhibited in cells upon miR-200c-3p-mimics treatment (both *P* < 0.05). In A498 cells, no significance was found in proportion of cells in G1, S, and G2 phases together with cell apoptosis in cells without treatment, cells transfected with miR-200c-3p-inhibitors NC sequence and miR-200c-3p-inhibitors + si-SOX2 sequence (*P* > 0.05). In cells introduced with miR-200c-3p-inhibitors, there showed fewer cells arrested at G1 phrase and more arrested in S phrase as well as declined apoptosis rate, while an opposite tread was found upon si-SOX2 treatment (both *P* < 0.05).

### Upregulation of miR-200c-3p and downregulation of SOX2 repress cell migration and invasion of RCC cells

**Fig. 8:**
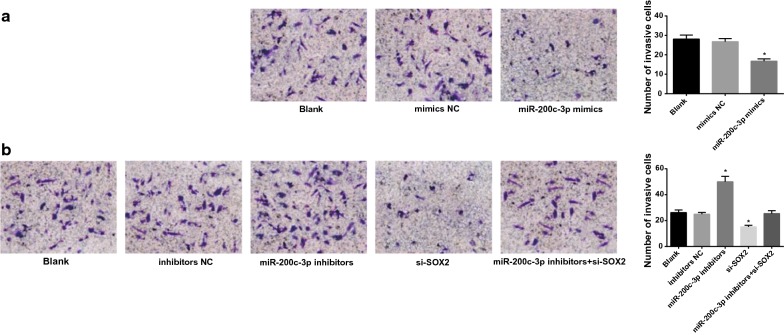
Upregulation of miR-200c-3p represses cell invasion of RCC cells. **a** Cell invasion after miR-200c-3p-mimics transfection into OS-RC-2 cells. **b** Cell invasion after miR-200c-3p-inhibitors and si-SOX2 transfection into A498 cells. N = 5; *P < 0.05 vs the blank group

**Fig. 9:**
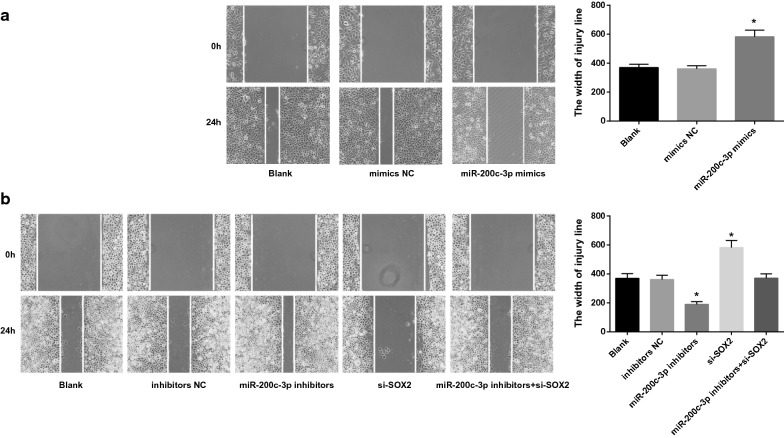
Upregulation of miR-200c-3p represses cell migration of RCC cells. **a** Cell migration after miR-200c-3p-mimics transfection into OS-RC-2 cells. **b** Cell migration after miR-200c-3p-inhibitors and si-SOX2 transfection into A498 cells. N = 5; *P < 0.05 vs the blank group

Transwell assay and scratch test (Figs. 8, 9) suggested that in OS-RC-2 cells, no difference was found in cell migration and invasion capabilities in cells without treatment and in cells transfected with miR-200c-3p-mimics NC sequence (*P* > 0.05). Cell migration and invasion abilities were inhibited in cells upon miR-200c-3p-mimics treatment (both *P* < 0.05). In A498 cells, no significance was found in cell migration and invasion capabilities in cells without treatment, cells transfected with miR-200c-3p-inhibitors NC sequence and miR-200c-3p-inhibitors + si-SOX2 sequence (*P* > 0.05). In cells transfected with miR-200c-3p-inhibitors, there showed enhanced cell migration and invasion abilities, while an opposite tread was found upon si-SOX2 treatment (both *P* < 0.05).

## Discussion

In recent years, the aberrant profiles RCC-specific miRNA have been discussed, while no consensus reached on the exact role of certain miRNAs in RCC [22–24]. Among all the miRNAs, the EMT-related miR-200 members have been often detected to be poorly expressed in RCC samples, demonstrating that these miRNAs could act as tumor suppressors in RCC [4]. For the purpose of improving the treatment of RCC, the molecular therapies are being widely applied for RCC patients with metastasis or recurrence. In view of this, we conduced this current study to unearth the function of miR-200c-3p in RCC with the involvement of SOX2-mediated Wnt/β-catenin signaling pathway. Collectively, the findings highlight that miR-200c-3p inhibits development of RCC cells via the SOX2-mediated Wnt/β-catenin signaling pathway.

One of this most important findings in this study implied that miR-200c-3p was reduced in RCC tissues and cell lines, and miR-200c-3p was implicated in the initiation and development of RCC. Besides, we also found that miR-200c-3p was determined to suppress proliferation, migration, invasion and induces apoptosis of RCC cells. It has been reported that the miR-200s modulates EMT-activating transcription factors and is downregulated in mesenchymal-like cancer cells through the regulation of TGF-β [25]. A recent study has elucidated that the miR-200s members could be transactivated by p53, and p53-modulated miRNAs are able to prevent EMT via binding to ZEB1 and ZEB2 [26]. Based on which, we could conclude that the miR-200s family, acting as a novel part of the p53 regulatory network, results in invasion and metastasis of human cancer cells via the EMT process. In accordance with the results in this current study, several articles have revealed that miR-200c is poorly expressed in RCC tissues [6–8]. Additionally, it was found that downregulated miR-200-3p was determined to suppress proliferation, migration, invasion and induces apoptosis of RCC cells. SOX2, defined as a main stemness marker, is upregulated in cancer stem cells, and it has the capability to produce the diversity of cell types [27]. Researches have strongly correlated SOX2 to cancer hallmarks, and SOX2 has been regarded to induce cellular proliferation (breast and cervical cancers) [28, 29]. However, the functional significance of miR-200c-3p and SOX2 expression in RCC remains to be elucidated.

Additionally, it was indicated that miR-200c-3p inhibited SOX2 to inactivate the Wnt/β-catenin signaling pathway. A study has suggested that miR-200a is a novel candidate target for tumor therapy through modulating the Wnt/β-catenin signaling pathway [30]. As previously reported, the Wnt/β-catenin signaling pathway was restricted by overexpressed miR-195-5p while was activated by suppressed miR-195-5p in RCC cells [31]. Meanwhile, the results in another study has demonstrated that UBE3C may have a relationship with proliferation, invasion as well as migration of RCC cells via the Wnt/β-catenin signaling pathway activation [21]. It has been revealed that an incapability to inhibit the Wnt/β-catenin signaling may result in the pathogenesis of loss-of-function mutations of SOX2 in human patients [32]. Evidence has shown that overexpression of SOX2 leads to increased expression of β-catenin [33], and overexpression of SOX2 activates the Wnt/β-catenin pathway [34]. However, it has also been suggested that overexpression of SOX2 contributed to suppression of Wnt/β-catenin signaling activity [35]. The inconsistent results might result from different tumor types combined with other influencing factors. Another study has indicated SOX15 repressed tumor formation in pancreatic ductal adenocarcinoma via the block of the Wnt/β-catenin signaling pathway [36]. We also found that SOX2 was a target gene of miR-200c-3p. In accordance with the results in our study, a prior study has revealed that miR-200c-3p downregulation results in the paclitaxel resistance of breast cancer cells through targeting SOX2 [37]. Another study has demonstrated that miR-200 family members regulating SOX2 and E2F3 might be of great importance to transfer pluripotent or multipotent stem/progenitor cells to more differentiated cells [38]. Furthermore, it has been suggested that miR-200c overexpression could downregulate SOX2 and KlLF4 and elevate the activity of Wnt signaling suppressed by SOX2, implying that miR-200c could function as a unique osteo-inductive agent used for bone healing and regeneration [39]. Liao et al. have found that novel interaction between SOX17 and miR-200 [40], and another study also suggested that SOX-1 and SOX-9 are determined to be the direct targets of miR-200 and miR-145, respectively [41]. The relationship between miR-200c-3p and SOX2 needs further verification. All these above verified the correlations among miR-200c-3p, SOX2 and the Wnt/β-catenin signaling pathway.

Our study also had advantage and limitation. This current study offers a effective and promising approach for miRNA-related and evidence-based RCC therapy, which could be helpful for the treatment of RCC in clinical. Nevertheless, due to the lack of follow-up data, this study failed to elucidate the role of the examined molecular markers as potential prognostic markers, which will be studies in future research.

## Conclusion

In conclusion, EMT-associated miR-200-3p were decreased in clinical RCC samples, which may act as a tumor inhibitor via binding to certain cancer-associated genes and pathways. Besides, the results from this current study could offer a effective and promising approach for miRNA-related and evidence-based RCC therapy. Thus, better recognition of the in-depth molecular pathways controlled by miR-200-3p might contribute to better diagnostic, therapeutic and prognostic as well as interventions for RCC. However, we have no relevant analysis for the calculation of the survival data, and we will conduct relevant experimental analysis to achieve better results in the follow-up experiments.

## Data Availability

Not applicable.

## Abbreviations

mir-200c-3p: microRNA-200c-3p
RCC: renal cell carcinoma
EMT: epithelial mesenchymal transition
SOX: SRY (sex determining region Y)-box
ATCC: American Type Culture Collection
FBSP: fetal bovine serum
RT-PCR: reverse transcription quantitative polymerase chain reaction
DEPC: diethylpyrocarbonate
ANOVA: analysis of variance
LSD-t: least significant difference t test
